# Acute high altitude exposure, acclimatization and re-exposure on nocturnal breathing

**DOI:** 10.3389/fphys.2022.965021

**Published:** 2022-09-05

**Authors:** Michael Furian, Konstantinos Bitos, Sara E. Hartmann, Lara Muralt, Mona Lichtblau, Patrick R. Bader, Jean M. Rawling, Silvia Ulrich, Marc J. Poulin, Konrad E. Bloch

**Affiliations:** ^1^ University Hospital Zurich, Department of Respiratory Medicine, Zurich, Switzerland; ^2^ University of Calgary, Cumming School of Medicine, Department of Physiology and Pharmacology and Hotchkiss Brain Institute, Calgary, AB, Canada; ^3^ University of Calgary, Cumming School of Medicine, Department of Family Medicine, Calgary, AB, Canada

**Keywords:** hypoxia, altitude (MeSH), respiration - physiology, sleep-disordered breathing, respiratory polygraphy

## Abstract

**Background:** Effects of prolonged and repeated high-altitude exposure on oxygenation and control of breathing remain uncertain. We hypothesized that prolonged and repeated high-altitude exposure will improve altitude-induced deoxygenation and breathing instability.

**Methods:** 21 healthy lowlanders, aged 18-30y, underwent two 7-day sojourns at a high-altitude station in Chile (4–8 hrs/day at 5,050 m, nights at 2,900 m), separated by a 1-week recovery period at 520 m. Respiratory sleep studies recording mean nocturnal pulse oximetry (SpO_2_), oxygen desaturation index (ODI, >3% dips in SpO_2_), breathing patterns and subjective sleep quality by visual analog scale (SQ-VAS, 0–100% with increasing quality), were evaluated at 520 m and during nights 1 and 6 at 2,900 m in the 1st and 2nd altitude sojourn.

**Results:** At 520 m, mean ± SD nocturnal SpO_2_ was 94 ± 1%, ODI 2.2 ± 1.2/h, SQ-VAS 59 ± 20%. Corresponding values at 2,900 m, 1st sojourn, night 1 were: SpO_2_ 86 ± 2%, ODI 23.4 ± 22.8/h, SQ-VAS 39 ± 23%; 1st sojourn, night 6: SpO_2_ 90 ± 1%, ODI 7.3 ± 4.4/h, SQ-VAS 55 ± 20% (*p* < 0.05, all differences within corresponding variables). Mean differences (Δ, 95%CI) in acute effects (2,900 m, night 1, vs 520 m) between 2nd vs 1st altitude sojourn were: ΔSpO_2_ 0% (-1 to 1), ΔODI -9.2/h (-18.0 to -0.5), ΔSQ-VAS 10% (-6 to 27); differences in acclimatization (changes night 6 vs 1), between 2nd vs 1st sojourn at 2,900 m were: ΔSpO_2_ -1% (-2 to 0), ΔODI 11.1/h (2.5 to 19.7), ΔSQ-VAS -15% (-31 to 1).

**Conclusion:** Acute high-altitude exposure induced nocturnal hypoxemia, cyclic deoxygenations and impaired sleep quality. Acclimatization mitigated these effects. After recovery at 520 m, repeated exposure diminished high-altitude-induced deoxygenation and breathing instability, suggesting some retention of adaptation induced by the first altitude sojourn while subjective sleep quality remained similarly impaired.

## Introduction

Recent developments in means of transport and infrastructure make traveling to high altitude increasingly common. Many settlements worldwide are located at high altitudes (above 2,500 m), with regular working places up to more than 5,000 m. Especially, astronomical observatories and mines are located at very high altitudes. Working schedules of professionals at such sites may require rapid ascent from low altitude and subsequent shifts of one or several days or repeated shifts of several days alternating with periods of recovery at low altitude. The effects of prolonged or repeated exposure to high altitude have not been extensively studied but may include discomfort and high-altitude illness (Luks et al., 2017). Even though physiological acclimatization tends to counteract the effects of the reduced inspired PO_2_ and consecutive hypoxemia, depending on the altitude reached and individual susceptibility, this response may not prevent adverse consequences or it may itself be perceived as uncomfortable. Thus, at altitudes >1,500 m, high altitude periodic breathing, an oscillatory pattern of waxing and waning of ventilation with periods of hyperventilation alternating with central apneas or hypopneas, is commonly observed in healthy subjects during sleep and sometimes also during wakefulness and even during physical exertion (Latshang et al., 2013a; Bloch et al., 2015). It may be associated with frequent arousals from sleep with a distressing sense of suffocation that prevents revitalizing rest and it may impair daytime performance (Latshang et al., 2013b). Although high altitude periodic breathing has been well known for many years (West et al., 1985) its evolution during a sojourn of a few days or during repeated sojourns at high altitude are incompletely understood. Moreover, the consequences of high altitude periodic breathing in terms of daytime performance in real working schedules of people at such altitudes remain elusive even though they are clinically highly relevant.

Therefore, the purpose of the current study was to evaluate in healthy volunteers the effects of high altitude on nocturnal oxygenation and the breathing pattern during acute exposure, during prolonged exposure with daily ascents to very high altitude, and during re-exposure. This pattern of altitude exposure was selected to mimic that of professionals in high altitude work places. We hypothesized, a), that acute high altitude exposure induces pronounced nocturnal hypoxemia and periodic breathing that improves over the course of a few days and nights at high altitude and that, b), re-exposure to high altitude after 1 week staying near sea level induces less pronounced hypoxemia and periodic breathing compared to the first exposure suggesting retention of some altitude-induced physiological changes.

## Methods

### Ethical approval

All participants gave written informed consent and the study was approved by the University of Calgary, Canada, Conjoint Health Research Ethic Board (CHREB ID: REB15-2709) and the Cantonal Ethic Committee Zurich, Switzerland (REQ-2016-00048). The trial was registered at ClinicalTrials.gov (NCT02730143).

### Study design and setting

**FIGURE 1:**
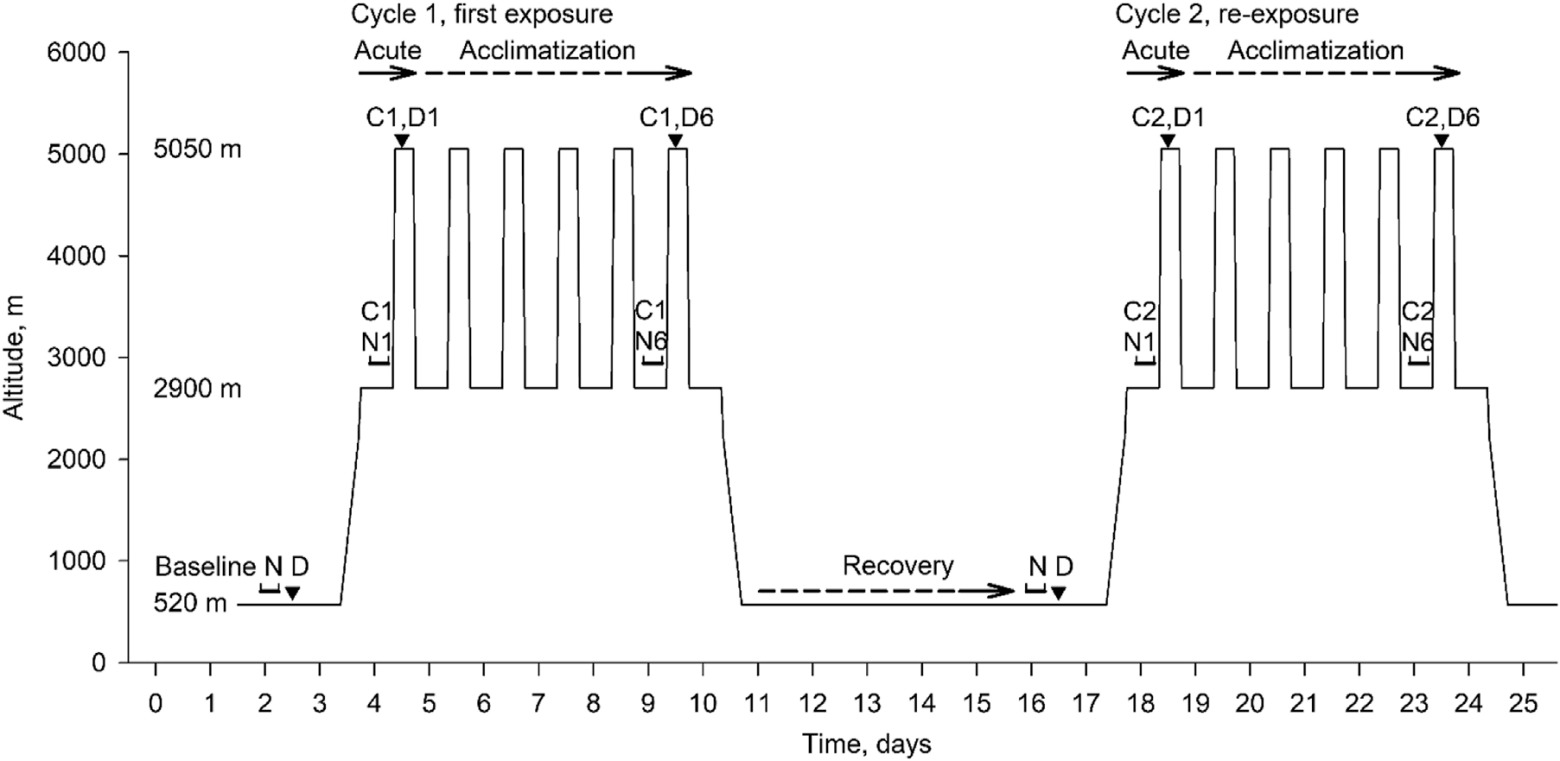
Overview of study design and data collection. The consecutive phases were: baseline period at 520 m, first high altitude sojourn (Cycle 1, days 4–10, with nights spent at 2,900 m, days at 5,050 m); recovery period at 520 m, days 11–17; second altitude sojourn (Cycle 2, days 18–24). Assessments included overnight sleep studies (N) and daytime assessments (D). Effects of acute high altitude exposure were evaluated in Cycle 1 during night 1 and day 1 (C1, N1, C1, D1) in comparison to baseline night and day (N D); effects of acclimatization in Cycle 1 were evaluated in night 6 and day 6 (C1, N6, C1, D6) in comparison to the first night and day. Corresponding evaluations took place during the high altitude re-exposure in Cycle 2. The acute effects of high altitude re-exposure were evaluated in Cycle 2 in comparison to overnight and daytime assessments at the end of the low altitude recovery period.

This prospective study carried out in Chile comprised 3 consecutive 7-day periods. Participants spent two 7-day cycles at high altitude and recovered for 7 days near sea level in-between (Figure 1). After baseline measurements in Santiago de Chile (LA, 520 m, 1,706 ft, barometric pressure 709 mmHg), participants travelled for 2 h by plane and 2 h by bus to the Atacama Large Millimeter-Submillimeter Array (ALMA) Operation Support Facility (ASF, 2,900 m, 9,514 ft, barometric pressure 542 mmHg) where they spent the next 7 nights. During daytime over this period, they travelled by car within 45 min to the ALMA Operation Site (AOS, 5,050 m, 16,568 ft, barometric pressure 419 mmHg) and stayed there for 4–8 h without oxygen supplementation. This exposure pattern is similar to that of workers at the telescope station. After the first 7-day cycle at high altitude, participants travelled back to 520 m for 7 days of recovery. A second high altitude sojourn, Cycle 2, with identical protocol as in Cycle 1 concluded the study. At 520 m and during the 1^st^ and 6^th^ night at 2,900 m, respiratory sleep studies were performed, followed by daytime evaluations in the morning at 2,900 m and additional evaluations over the course of the day at 5,050 m as illustrated in Figure 1. The study was performed in conjunction with examinations on cognitive performance published previously (Pun et al., 2018a; Pun et al., 2018b). Participant characteristics and reaction time have been presented in the cited reports. Data from sleep studies, the focus of the current paper, have not been published.

### Participants

Healthy men and women were recruited in the area of Calgary, Canada, and Zurich, Switzerland. Exclusion criteria included a previous history of intolerance to altitudes <3,000 m, current pregnancy, or any health impairments which required regular treatment. All participants underwent clinical examinations prior to altitude sojourns. During the expedition, caffeine consumption was allowed while alcohol or any medication (in particular acetazolamide, among others) were prohibited.

### Measurements

Prior to the baseline measurement at 520 m, a familiarization session was performed in the preceding day/night in all participants.

#### Respiratory sleep studies

Recordings were performed from 22:00 to 06:00 (time in bed, TIB). Measurements included finger pulse oximetry (SpO_2_), nasal cannula pressure swings, thoracic and abdominal excursions by inductance plethysmography, electrocardiogram (ECG) and body position (AlicePDx, Philips AG Respironics, Zofingen, Switzerland). Breathing disturbances were scored as reported previously (AASM, 1999; Bloch et al., 2010). Apnea/hypopnea were defined as a >50% reduction in nasal pressure swings or chest wall excursions for ≥10 s. Obstructive apneas/hypopneas were scored if asynchronous or paradoxical chest wall excursions suggested continued effort during the event or if a flattened inspiratory portion of the nasal pressure curve suggested flow limitation. Central apneas/hypopneas were scored in the absence of criteria of obstructive events. Three or more consecutive central apneas/hypopneas with a duration of ≥5 s were scored as periodic breathing. The apnea/hypopnea index (AHI) was defined as the number of events/h TIB, the oxygen desaturation index (ODI) as the number of SpO_2_ desaturation dips >3%/h TIB.

#### Clinical examination, questionnaires and spirometry

At 2,900 m, blood pressure, pulse rate, weight and general well-being were evaluated before breakfast in the morning after sleep studies. Participants rated their sleepiness by the Karolinska Sleepiness Scale ranging from 1 (very awake) to 9 (very tired) (Kaida et al., 2006). Subjective sleep quality was assessed by a visual analog scale ranging from 0 (extremely bad) to 100 mm (excellent). Insomnia was assessed by asking participants to estimate the time until falling asleep, the number of awakenings and total time spent awake during the night.

Daytime assessments at 5,050 m included spirometries with reference values of the Global Lung Function Initiative (Quanjer et al., 2012), clinical examinations and assessment of sniff inspiratory nasal pressure (SNIP) (Laveneziana et al., 2019) after several hours exposure to very high altitude.

### Outcomes

The main outcome was the mean nocturnal SpO_2_ over the course of high altitude exposure during two sojourns at 2,900 m compared to 520 m. Secondary outcomes were further indices of oxygenation and of high altitude periodic breathing such as AHI, ODI and results from clinical assessments, sleep-related questionnaires and lung function.

### Sample size

To detect a minimal difference in nocturnal SpO_2_ of 2% (SD of 3%) with a power of 80%, alpha level of 0.05 and dropout rate of 10%, 21 participants were required. This sample size allowed to detect changes in important secondary outcomes such as difference in AHI or ODI between acute exposures to high altitude in Cycle 2 vs Cycle 1 of 10 events/h, assuming a SD of 15 events/h.

### Statistical analysis

According to the intention-to-treat principle, missing data in the primary outcome (SpO_2_) were replaced by multiple imputations (n = 20) using regression models with chained equations including anthropometrics, altitude location, Cycle and examination day as independent predictors (Zhang, 2016). Occasional missing data in secondary outcomes were not replaced. Data are presented as means ± SD. Mean differences and 95% confidence intervals were computed using mixed linear regression models with outcomes as dependent variable and altitude location, Cycle and examination day as independent variables. For the primary outcome SpO_2_, Bonferroni corrections to account for 2 comparisons (differences between 2nd vs 1st Cycle in acute altitude effects and acclimatization effects) were applied. For secondary outcomes, only hypothesis-based comparisons were performed to minimize the false positive discovery rate. Statistical significance was assumed at *p* < 0.05 and if 95% confidence intervals of mean differences did not include zero.

## Results

**FIGURE 2:**
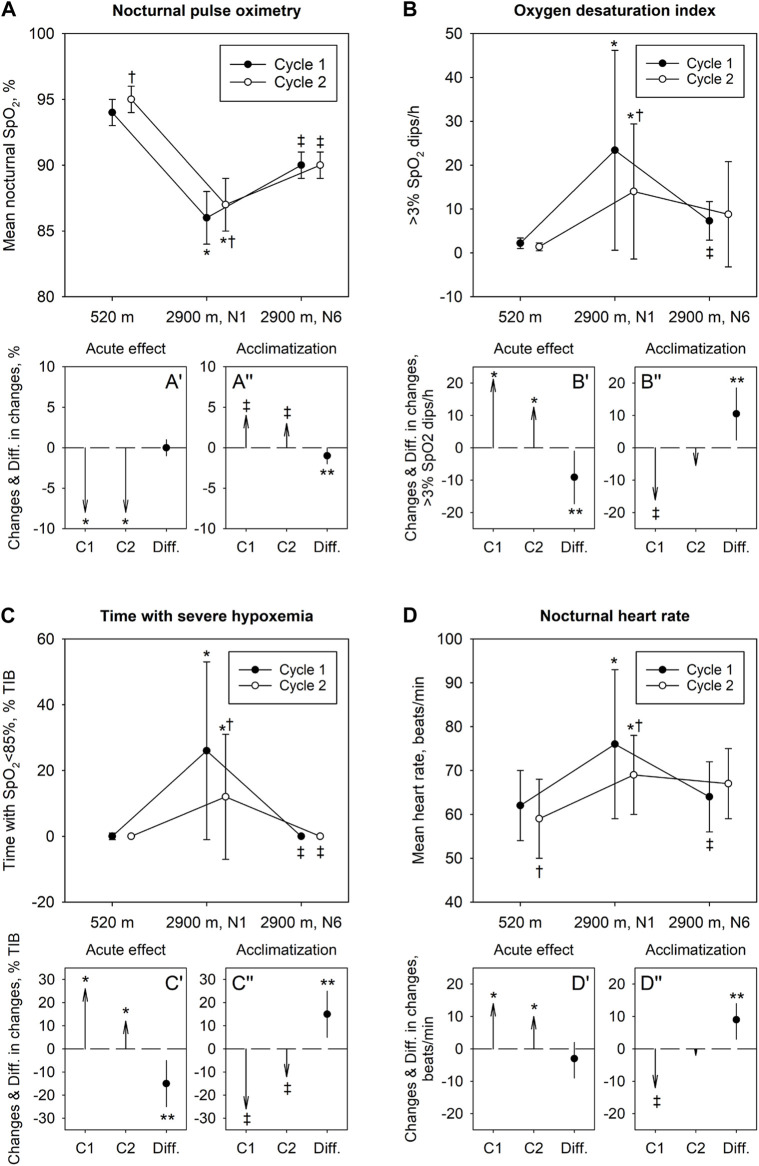
Effect of acute high-altitude exposure, acclimatization and re-exposure on indices of nocturnal oxygenation **(A–C)** and heart rate **(D)**. In panels **(A–D)** mean ± SD values at 520 m and at 2,900 m, nights 1 and 6, in the Cycle 1 and 2 are shown. Panels A′-D′ illustrate changes in variables with acute ascent in Cycle 1 and 2 (vector arrows C1 and C2) along with their mean difference (Diff.) and 95% confidence interval. Panels A″-D″ illustrate changes with acclimatization in Cycle 1 and 2 along with their mean difference and 95% confidence interval. *p < 0.05 vs 520 m in corresponding Cycle (acute altitude effect); ‡*p* < 0.05 vs 1^st^ night at 2,900 m in corresponding Cycle (acclimatization effect). †*p* < 0.05 vs corresponding Baseline or value in Cycle 1, respectively (repeated exposure effect); **p < 0.05 for differences between Cycles.

All 21 healthy volunteers completed the study. Baseline characteristics are presented in Table 1. No adverse events requiring therapeutic interventions were reported. Data from all 21 participants were analyzed. The results are displayed in Figure 2 (individual trends in Supplementary Figure S1) and numerically reported in Tables 2 and 3.

**TABLE 1:** Participant characteristics.

N (men/women)	21 (8/13)
Age, years	25 (21; 28)
Weight, kg	66.5 (56.0; 73.5)
Height, m	1.71 (1.63; 1.75)
Body Mass Index, kg/m^2^	21.5 (21.0; 24.1)

Values are counts and medians (quartiles).

**TABLE 2:** Sleep studies and subjective sleep assessment.

	Baseline	Cycle 1		Recovery	Cycle 2		Mean difference (95%CI) in acute altitude effects between cycles 1 and 2	Mean difference (95%CI) in acclimatization effects between cycles 1 and 2
	520 m	2,900 m, 1st night	2,900 m, 6th night	520 m	2,900 m, 1st night	2,900 m, 6th night		
Sleep study results								
Time in bed, min	463 ± 31	466 ± 41	482 ± 22	464 ± 86	470 ± 66	466 ± 76	6 (−35 to 46)	−22 (−62 to 18)
Mean nocturnal SpO_2_, %	94 ± 1	86 ± 2*	90 ± 1‡	95 ± 1†	87 ± 2*,†	90 ± 1‡	0 (−1 to 1)	−1 (−2 to 0)**
SpO_2_ <95%, % time in bed	46 ± 30	99 ± 2*	98 ± 3	27 ± 25†	97 ± 7*	98 ± 2	18 (1 to 34)**	1 (−15 to 17)
SpO_2_ <90%, % time in bed	1 ± 2	88 ± 14*	47 ± 25‡	0 ± 1	83 ± 18*	45 ± 27‡	−4 (−17 to 8)	3 (−10 to 15)
SpO_2_ <85%, % time in bed	0 ± 1	26 ± 27*	0 ± 0‡	0 ± 0	12 ± 19*,†	0 ± 0‡	−15 (−25 to −5)**	15 (5 to 25)**
Oxygen desaturation index, 1/h	2.2 ± 1.2	23.4 ± 22.8*	7.3 ± 4.4‡	1.4 ± 0.9	14.0 ± 15.4*,†	8.8 ± 12.0	−9.2 (−18 to −0.5)**	11.1 (2.5 to 19.7)**
Apnea/hypopnea index, 1/h	8.0 ± 5.2	26.6 ± 29.4*	17.4 ± 20.8‡	7.1 ± 5.3	20.2 ± 22.5*	14.1 ± 15.3	−6.5 (−18.2 to 5.2)	3.9 (−7.7 to 15.6)
Central AHI, 1/h	1.0 ± 1.0	18.1 ± 29.7*	9.5 ± 19.9‡	0.9 ± 0.9	12.9 ± 21.6*	7.9 ± 15.0	−6.1 (−18 to 5.9)	4.4 (−7.4 to 16.3)
Obstructive AHI, 1/h	7.0 ± 5.1	8.6 ± 7.0*	7.9 ± 4.6	6.1 ± 4.9	7.3 ± 3.6	6.2 ± 3.0	−0.4 (−3.3 to 2.5)	−0.4 (−3.3 to 2.4)
Periodic breathing, min	0 ± 0	44 ± 79*	25 ± 60	0 ± 0	32 ± 60*	18 ± 43	−14 (−47 to 20)	7 (−27 to 40)
Mean nocturnal heart rate, 1/min	62 ± 9	76 ± 17*	64 ± 8‡	59 ± 9†	69 ± 9*,†	67 ± 8	−3 (−9 to 2)	9 (3 to 14)**
Subjective assessments in the morning after sleep studies								
Karolinska sleepiness score	5 ± 2	5 ± 2	4 ± 2	5 ± 2	5 ± 2	5 ± 2	0 (−1 to 2)	0 (−1 to 1)
Estimated time to falling asleep, min	21 ± 23	45 ± 61*	25 ± 20	44 ± 59†	28 ± 25	43 ± 43	−41 (−72 to −10)**	36 (5 to 67)**
Estimated awakenings at night, n	4 ± 6	5 ± 5	2 ± 3‡	3 ± 3	4 ± 4	4 ± 7	0 (−3 to 2)	3 (0 to 6)**
Estimated time spent awake at night, min	29 ± 27	51 ± 55*	22 ± 18‡	34 ± 30	39 ± 28	68 ± 53‡^,^†	−16 (−46 to 13)	58 (29 to 87)**
Subjective sleep quality, %VAS	59 ± 20	39 ± 23*	55 ± 20‡	53 ± 21	44 ± 26*	44 ± 21	10 (−6 to 27)	−15 (−31 to 1)

Means ± SD, and mean differences (95% confidence intervals), n = 21. *p < 0.05 vs Baseline at 520 m (acute altitude effect); ‡*p* < 0.05 vs 1st night at 2,900 m in the corresponding Cycle (acclimatization effect). †*p* < 0.05 vs corresponding value in Baseline and Cycle 1, respectively (repeated exposure effect); **p < 0.05 for differences between Cycles in acute altitude effects and acclimatization. SpO_2_ = pulse oximetry; VAS, Subjective sleep quality assessed by a visual analog scale ranging from 0 (very bad) to 100% (excellent).

**TABLE 3:** Daytime assessments at 520 m, 2,900 m and 5,050 m.

	Baseline	Cycle 1		Recovery	Cycle 2		Mean difference (95%CI) in acute altitude effects between cycles 1 and 2	Mean difference (95%CI) in acclimatization effects between cycles 1 and 2
	520 m	2,900 m, 1st day	2,900 m, 6th day	520 m	2,900 m, 1st day	2,900 m, 6th day		
Clinical evaluation and assessment at 2,900 m								
Heart rate, 1/min	61 ± 10	79 ± 18*	64 ± 11‡	57 ± 9†	66 ± 10†	64 ± 9	−8 (−15 to −2)**	13 (7 to 20)**
Mean blood pressure, mmHg	79 ± 9	79 ± 10	77 ± 8	75 ± 8†	81 ± 9	78 ± 9	5 (0 to 11)	−1 (−6 to 5)
Spirometry and pulse oximetry at 5,050 m								
FVC, L	4.4 ± 0.8	4.1 ± 0.7*	4.2 ± 0.7	4.4 ± 0.8	4.1 ± 0.7*	4.2 ± 0.8	−0.1 (−0.2 to 0.0)	0.1 (−0.1 to 0.2)
FVC, %predicted	94 ± 12	91 ± 11*	93 ± 10	94 ± 14	92 ± 10*	91 ± 13	−2 (−5 to 1)	1 (−2 to 4)
FEV_1_, L	3.7 ± 0.6	3.6 ± 0.6	3.7 ± 0.5	3.6 ± 0.6	3.7 ± 0.5	3.7 ± 0.6	0.1 (−0.1 to 0.2)	0.0 (−0.2 to 0.1)
FEV_1_, %predicted	96 ± 11	96 ± 11	99 ± 10	95 ± 11	99 ± 9	98 ± 13	2 (−1 to 5)	−1 (−4 to 3)
FEV_1_/FVC	0.85 ± 0.06	0.88 ± 0.06*	0.88 ± 0.05	0.84 ± 0.07	0.89 ± 0.06*,†	0.89 ± 0.06	0.03 (0.01 to 0.05)**	−0.02 (−0.04 to 0.00)
SNIP, mmHg	106 ± 29	88 ± 22*	89 ± 28	108 ± 35	90 ± 27*	96 ± 30†	−1 (−11 to 9)	8 (−2 to 18)

Values are in means ± SD. *p < 0.05 vs 520 m in the corresponding Cycle (acute altitude effect); ‡*p* < 0.05 vs 1^st^ night at 2,900 m in the corresponding Cycle (acclimatization effect). †*p* < 0.05 vs corresponding value in Baseline and Cycle 1, respectively (repeated exposure effect); **p < 0.05 for differences between Cycles in acute altitude effects and acclimatization. FEV_1_ = forced expiratory volume in 1 s; FVC, forced vital capacity; SNIP, sniff nasal inspiratory pressure.

### Respiratory sleep studies

Among 126 assessments of the main outcome, missing data had to be replaced by multiple imputations in 3 instances (2%). Baseline sleep studies revealed normal indices of nocturnal oxygenation and a normal AHI. In the first night at 2,900 m (Cycle 1, night 1), there was a significant decrease in nocturnal oxygenation reflected in lower mean nocturnal SpO_2_ and longer night-time spent with SpO_2_ <95%, <90% and <85%. Compared to 520 m, the ODI and AHI were increased in the first night at 2,900 m related to emergence of periodic breathing with central apneas/hypopneas. After 5 additional nights at 2,900 m (Cycle 1, night 6), SpO_2_, heart rate, ODI and AHI had partially normalized (*p* < 0.05 Cycle 1, night 6 vs night 1, all comparisons). At the end of the recovery period at 520 m, the second baseline sleep studies revealed a higher nocturnal SpO_2_ and a lower heart rate compared to the first baseline.

After 7 nights at 520 m, during re-exposure to high altitude in Cycle 2, the sleep study in the first night at 2,900 m (Cycle 2, night 1) again revealed decreased pulse-oximetric indices of oxygenation and increased ODI and heart rate (*p* < 0.05 compared to 520 m) but these changes, although similar compared to Cycle 1, resulted in less altitude-induced deteriorations (Table 2). After 5 nights in Cycle 2 at 2,900 m, SpO_2_ and ODI had partially improved but the changes were less pronounced than those in the Cycle 1 as the initial deviations of the values from low altitude baseline were less prominent than those in Cycle 1, night 1 (Figure 2, Supplementary Figure S1).

### Subjective sleep assessment

Compared to low altitude baseline, the first acute exposure to 2,900 m was associated with a reduced subjective sleep quality, a longer estimated time to fall asleep and a longer time spent awake during the night. These changes partially improved with acclimatization over 5 days (Table 2). After returning to 520 m, subjective sleep quality, estimated time spent awake and number of awakenings were similar as those in the first baseline night at 520 m.

In Cycle 2, the estimated time to fall asleep in night 1 was significantly shorter than that in the Cycle 1, night 1. In Cycle 2, the estimated time spent awake was increased in night 5 compared to night 1 and the time to fall asleep, the number of awakenings and subjective sleep quality did not change over the course of Cycle 2.

### Daytime evaluation

Ascent to 2,900 m and 5,050 m in Cycle 1 was associated with an increase in heart rate, a reduction in forced vital capacity (FVC) and SNIP in Cycle 1, on day 1 at 5,050 m. These values did not significantly change over the altitude sojourn in Cycle 1.

In Cycle 2 at 5,050 m, heart rate and blood pressure remained unchanged but reductions in FVC and SNIP were noted in comparison to the second baseline. Over the course of the altitude sojourn, daytime assessments revealed no significant changes in heart rate, blood pressure and lung function.

## Discussion

We performed a comprehensive prospective clinical and physiological study in young, healthy volunteers who spent two periods (Cycles) of 7 nights at 2,900 m combined with daytime sojourns at 5,050 m, with a 7-days recovery period near sea level in-between. Compared to baseline near sea level, the main findings during the first Cycle at high altitude included pronounced nocturnal hypoxemia and periodic breathing associated with impaired subjective sleep quality after arrival at 2,900 m. These acute altitude-effects partially improved with acclimatization over the course of the stay at high altitude. After low-altitude recovery, evaluations during the second high-altitude Cycle revealed less pronounced acute physiological and subjective effects suggesting some retention of adaptation induced by the first altitude sojourn. Our findings are novel and clinically important as they may help to optimize initial and longer term work schedules and the use of measures (such as oxygen or medication, for example) that reduce adverse effects of altitude in professionals at high altitude work places.

### Acute altitude exposure (cycle 1)

It has been previously shown that acute exposure to high altitude is associated with nocturnal hypoxemia, periodic breathing, impaired sleep quality and frequent awakenings (Nussbaumer-Ochsner et al., 2012a; Latshang et al., 2013b; Bloch et al., 2015). Periodic breathing is promoted by hypoxemia that increases the gain of the respiratory feed-back control system by enhancing the hypoxic and hypercapnic ventilatory response and a by reducing the CO_2_ reserve (Lahiri et al., 1983; Dempsey, 2005; Kohler et al., 2008; Nussbaumer-Ochsner et al., 2012b; Nussbaumer-Ochsner et al., 2012c). In 51 healthy men, who stayed overnight at 2,590 m, we observed mild hypoxemia and periodic breathing (median SpO_2_ 90%, AHI 13.1/h) associated with a slight (3%) reduction of deep non-rapid eye movement sleep stages (NREM 3 and 4), slow wave power and a slight increase in arousals but no impairment in daytime psychomotor vigilance (PVT) reaction time (Latshang et al., 2013b). In 16 mountaineers studied at a higher altitude of 4,559 m, hypoxemia and periodic breathing were more pronounced (median SpO_2_ 67%, AHI 60.9/h) and the proportion of deep sleep was reduced more (reduction of NREM 3 and 4 by 12% vs sea level) than at 2,590 m. In the current study, nocturnal SpO_2_ (86%) and the AHI (26.6/h) were between the values reported at 2,590 m and 4,559 m illustrating the altitude-dependence of hypoxemia and breathing disturbances. Because of logistic constraints, neurophysiologic monitoring sleep was not feasible in the current study. Therefore, we are unable to assess potential interactions among breathing and sleep disturbances that may have modified the observed changes in AHI. Expectedly, the altitude-induced rise in AHI was predominantly due to central apneas/hypopneas but a slight increase in obstructive events was also observed (Table 2). We propose that the mechanisms promoting high altitude periodic breathing mentioned above may also have enhanced the propensity of certain subjects with sleep-induced upper airway instability to experience some obstructive events as shown previously at lower altitude (Latshang et al., 2013b).

In the current study, subjectively perceived sleep quality assessed by VAS was impaired during the first night at 2,900 m (Table 2). Presumably, sleep disruption and hypoxemia at high altitude account for the impaired daytime vigilance and cognitive performance reported previously in the participants of the current study (Pun et al., 2018a; Pun et al., 2018b) but this was not associated with subjectively perceived sleepiness. Altitude exposure may therefore have differential effects on perceived sleep quality, sleepiness and cognitive performance and/or the instruments used to evaluate these outcomes may vary in their responsiveness to effects of hypoxia. Spirometry at 5,050 m revealed a reduced FVC in association with a reduced SNIP suggesting an altitude-related reduction in inspiratory muscle strength and, possibly, interstitial pulmonary fluid accumulation as reported previously (Clarenbach et al., 2012). These changes may alter the plant gain of the respiratory feed-back control system and thereby modulate the propensity to periodic breathing (Dempsey, 2005) although our data do not allow corroboration of this hypothesis. The higher forced expiratory volume in 1 s/FVC ratio at 5,050 m compared to near sea level is consistent with a reduced air density and an associated reduction in airflow resistance.

### Acclimatization to high altitude (cycle 1)

The time course of changes in ventilatory control induced by a prolonged stay at high altitude has not been extensively studied. While some studies found an increase of periodic breathing during high altitude acclimatization despite improving oxygenation (Salvaggio et al., 1998; Bloch et al., 2010; Nussbaumer-Ochsner et al., 2012a; Burgess et al., 2013), others have shown no change or a decrease in periodic breathing (Latshang et al., 2013b; Tseng et al., 2015). This variation has been suggested to depend on altitude, i.e., the degree of hypoxia (Ainslie et al., 2013; Bloch et al., 2015). Thus, in 34 mountaineers who climbed from 3,750 m to 7,546 m within 19–20 days, periodic breathing continuously increased even though SpO_2_ improved over the same period (Bloch et al., 2010). Conversely, in the current study, nocturnal SpO_2_, AHI and subjective sleep quality were significantly improved after spending 6 nights at 2,900 m and days at 5,050 m, supporting a positive role of acclimatization to these two alternating hypoxic stimuli in stabilizing breathing pattern and sleep quality at 2,900 m. The relative importance of the degree and the pattern of hypoxic stimuli experienced during wakefulness and sleep on changes in control of breathing during a prolonged stay at high altitude requires further study. As shown in our previous report (Pun et al., 2018a), the reduced PVT reaction time after acute exposure to 5,050 m improved as well with acclimatization supporting a role of nocturnal oxygenation and breathing pattern in next day cognitive performance.

### Re-exposure to high altitude (cycle 2)

During re-exposure to 2,900 m we observed milder acute altitude-effects on SpO_2_, in particular on indices of severe deoxygenation, and ODI compared to Cycle 1. This suggests, that physiologic acclimatization achieved during Cycle 1 was partially retained during low altitude recovery until beginning of the second altitude ascent, thereby mitigating acute effects of re-exposure to hypoxia. The milder acute altitude effects during re-exposure might be due to higher ventilatory responsiveness to CO_2_ and, thus, improved oxygenation during re-exposure as suggested by data obtained in 21 lowlanders re-exposed to 5,260 m after a previous stay at that altitude and a 16 days sojourn near sea level (Fan et al., 2014). CO_2_ responsiveness was not measured in our study, but the higher SpO_2_ after acclimatization is consistent with this assumption. Since acute, altitude-induced changes in spirometry and SNIP were similar in the 1st and 2nd Cycle (Table 3) we have no evidence that differences in periodic breathing between the Cycles were due to differences in plant gain of the respiratory feed-back control system. Acclimatization effects during the stay at high altitude in Cycle 2 were less pronounced than that during Cycle 1, which can be explained due to the milder acute altitude effects and a ceiling or floor effect in certain variables such as SpO_2_ and percent time spent with periodic breathing, for example, which revealed less pronounced deviation from normal values in the beginning of Cycle 2 and could therefore not improve further to the same extent as in Cycle 1. Subjective sleep quality in the first night at 2,900 m in Cycle 2 was similar compared to 2,900 m in Cycle 1, however, estimated time falling asleep improved during Cycle 2 suggesting some improvements in the altitude-related sleep latency observed in Cycle 1.

### Limitations

Study participants were young, healthy and active individuals and may not be representative for high altitude workers of older age or with pre-existing illness at the altitude of the ALMA or at other elevations. Whether one or several additional successive high/low altitude cycles or different lengths of exposure and break would optimize respiratory acclimatization requires further studies. As we did not assess sleep by neurophysiological monitoring we report nocturnal respiratory events in reference to time in bed. This may have resulted in some underestimation of AHI, ODI and of altitude effects on these variables compared to corresponding indices referenced to total sleep time since altitude is known to reduce sleep efficiency (Bloch et al., 2015).

## Conclusion

We showed that acclimatization and a repeated exposure to high altitude simulating a typical work/leisure schedule of professionals at high altitude mitigated acute physiological changes in terms of hypoxemia and cyclic deoxygenations during sleep due to partial retention of acclimatization achieved during a preceding altitude sojourn. These findings, combined with the improved cognitive performance observed during a second altitude sojourn in the same participants (Pun et al., 2018a; Pun et al., 2018b), indicate that the working schedule (‘7 days sleeping high, working higher’) and a second high altitude sleep/working schedule after a 7-day recovery period at near sea level reduce adverse effects of extreme altitude.

## Data Availability

The raw data supporting the conclusions of this article will be made available by the authors, without undue reservation.
