# Supplementary material for: Acute high altitude exposure, acclimatization and re-exposure on nocturnal breathing
Source: Front Physiol. 2022 Sep 5;13:965021. doi: 10.3389/fphys.2022.965021 (PMC9483161; doi:10.3389/fphys.2022.965021)
Supplement: Supplementary file 1 [file DataSheet1.PDF]

Supplement

-

Acute High Altitude Exposure, Acclimatization and Re-exposure on Nocturnal Breathing

Short title **Repeated altitude sojourns and nocturnal breathing**

Michael Furian[1], Konstantinos Bitos[1], Sara E. Hartmann[2], Lara Muralt[1], Mona Lichtblau[1], Patrick R. Bader[1], Jean M. Rawling[3], Silvia Ulrich[1], Marc J. Poulin[2], Konrad E. Bloch[1]

[1] University Hospital Zurich, Department of Respiratory Medicine, Zurich, Switzerland

[2] University of Calgary, Cumming School of Medicine, Dept. of Physiology & Pharmacology and Hotchkiss Brain Institute, Calgary, Canada

[3] University of Calgary, Cumming School of Medicine, Dept. of Family Medicine, Calgary, Canada

Correspondence

Prof. Dr. Konrad E. Bloch,

University Hospital Zurich, Department of Respiratory Medicine

Raemistreet 100, 8091 Zurich, Switzerland

Phone: +41 44 255 38 28, Fax: +41 44 255 44 51

Email: konrad.bloch@usz.ch

Conflict of interest: none

Figure S1: Effect of acute high-altitude exposure, acclimatization and re-exposure on indices of nocturnal oxygenation (panels A-C) and heart rate (panel D). In panels A-D mean ±SD values at 520 m and at 2900 m, nights 1 and 6, in the Cycle 1 and 2 are shown. Panels A'-D' illustrate changes in variables with acute ascent in Cycle 1 and 2 (vector arrows C1 and C2) along with their mean difference (Diff.) and 95% confidence interval. Panels A''-D'' illustrate changes with acclimatization in Cycle 1 and 2 along with their mean difference and 95% confidence interval. *P <0.05 vs. 520 m in corresponding Cycle (acute altitude effect); ‡P <0.05 vs. 1st night at 2900 m in corresponding Cycle (acclimatization effect). †P<0.05 vs. corresponding Baseline or value in Cycle 1, respectively (repeated exposure effect); **P<0.05 for differences between Cycles.

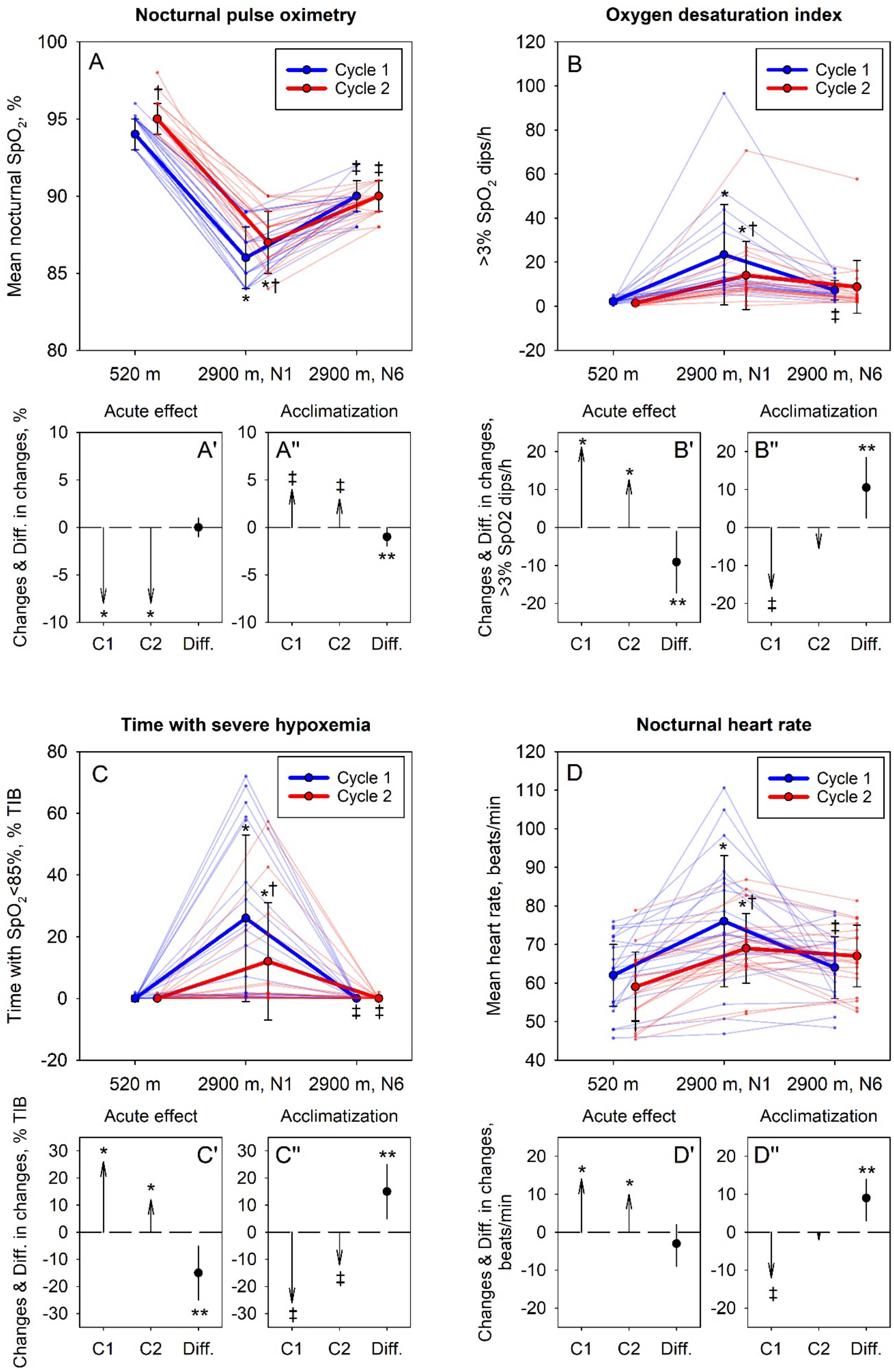
Nocturnal pulse oximetry
Oxygen desaturation index
Time with severe hypoxemia
Nocturnal heart rate
Cycle 1
Cycle 2
Mean nocturnal SpO2, %
>3% SpO2 dips/h
Time with SpO2<85%, % TIB
Mean heart rate, beats/min
520 m
2900 m, N1
2900 m, N6
Acute effect
Acclimatization
Changes & Diff. in changes, %
Changes & Diff. in changes, >3% SpO2 dips/h
Changes & Diff. in changes, % TIB
Changes & Diff. in changes, beats/min
C1
C2
Diff.

Figure S1
